# The liver–alpha cell axis associates with liver fat and insulin resistance: a validation study in women with non-steatotic liver fat levels

**DOI:** 10.1007/s00125-020-05334-x

**Published:** 2020-12-04

**Authors:** Christina Gar, Stefanie J. Haschka, Stefanie Kern-Matschilles, Barbara Rauch, Vanessa Sacco, Cornelia Prehn, Jerzy Adamski, Jochen Seissler, Nicolai J. Wewer Albrechtsen, Jens J. Holst, Andreas Lechner

**Affiliations:** 1 Diabetes Research Group, Department of Medicine IV, University Hospital, LMU Munich, Munich, Germany; 2 Clinical Cooperation Group Type 2 Diabetes, Helmholtz Zentrum München, Neuherberg, Germany; 3 German Center for Diabetes Research (DZD), Neuherberg, Germany; 4 Research Unit Molecular Endocrinology and Metabolism, Genome Analysis Center, Helmholtz Zentrum München, German Research Center for Environmental Health, Neuherberg, Germany; 5 Department of Biochemistry, Yong Loo Lin School of Medicine, National University of Singapore, Singapore, Singapore; 6 Chair of Experimental Genetics, Technical University of Munich, Freising-Weihenstephan, Germany; 7 Department of Biomedical Sciences, Faculty of Health and Medical Sciences, University of Copenhagen, Copenhagen, Denmark; 8 Department of Clinical Biochemistry, Rigshospitalet, Copenhagen, Denmark; 9 Novo Nordisk Foundation (NNF) Center for Protein Research, Faculty of Health and Medical Sciences, University of Copenhagen, Copenhagen, Denmark; 10 Novo Nordisk Foundation (NNF) Center for Basic Metabolic Research, Faculty of Health and Medical Sciences, University of Copenhagen, Copenhagen, Denmark

**Keywords:** Alanine/blood, Amino acids/blood, Cross-sectional studies, Female, Glucagon, Humans, Insulin resistance, Insulin resistance/physiology, Liver/metabolism, Liver–alpha cell axis

## Abstract

**Aims/hypothesis:**

Many individuals who develop type 2 diabetes also display increased glucagon levels (hyperglucagonaemia), which we have previously found to be associated with the metabolic syndrome. The concept of a liver–alpha cell axis provides a possible link between hyperglucagonaemia and elevated liver fat content, a typical finding in the metabolic syndrome. However, this association has only been studied in individuals with non-alcoholic fatty liver disease. Hence, we searched for a link between the liver and the alpha cells in individuals with non-steatotic levels of liver fat content. We hypothesised that the glucagon–alanine index, an indicator of the functional integrity of the liver–alpha cell axis, would associate with liver fat and insulin resistance in our cohort of women with low levels of liver fat.

**Methods:**

We analysed data from 79 individuals participating in the Prediction, Prevention and Subclassification of Type 2 Diabetes (PPSDiab) study, a prospective observational study of young women at low to high risk for the development of type 2 diabetes. Liver fat content was determined by MRI. Insulin resistance was calculated as HOMA-IR. We conducted Spearman correlation analyses of liver fat content and HOMA-IR with the glucagon–alanine index (the product of fasting plasma levels of glucagon and alanine). The prediction of the glucagon–alanine index by liver fat or HOMA-IR was tested in multivariate linear regression analyses in the whole cohort as well as after stratification for liver fat content ≤0.5% (*n* = 39) or >0.5% (*n* = 40).

**Results:**

The glucagon–alanine index significantly correlated with liver fat and HOMA-IR in the entire cohort (*ρ* = 0.484, *p* < 0.001 and *ρ* = 0.417, *p* < 0.001, respectively). These associations resulted from significant correlations in participants with a liver fat content >0.5% (liver fat, *ρ* = 0.550, *p* < 0.001; HOMA-IR, *ρ* = 0.429, *p* = 0.006). In linear regression analyses, the association of the glucagon–alanine index with liver fat remained significant after adjustment for age and HOMA-IR in all participants and in those with liver fat >0.5% (*β* = 0.246, *p* = 0.0.23 and *β* = 0.430, *p* = 0.007, respectively) but not in participants with liver fat ≤0.5% (*β* = −0.184, *p* = 0.286).

**Conclusions/interpretation:**

We reproduced the previously reported association of liver fat content and HOMA-IR with the glucagon–alanine index in an independent study cohort of young women with low to high risk for type 2 diabetes. Furthermore, our data indicates an insulin-resistance-independent association of liver fat content with the glucagon–alanine index. In summary, our study supports the concept that even lower levels of liver fat (from 0.5%) are connected to relative hyperglucagonaemia, reflecting an imminent impairment of the liver–alpha cell axis.
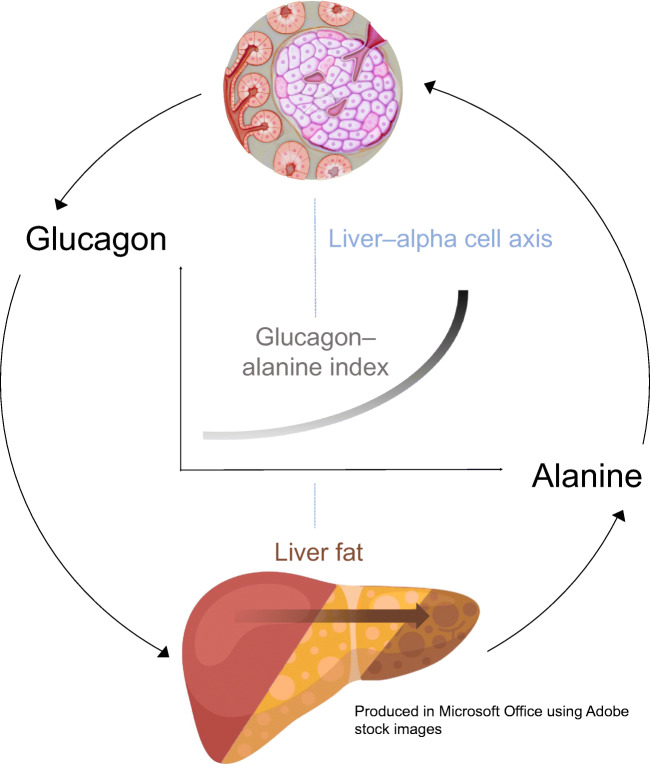

**Supplementary Information:**

The online version contains peer-reviewed but unedited supplementary material available at 10.1007/s00125-020-05334-x.

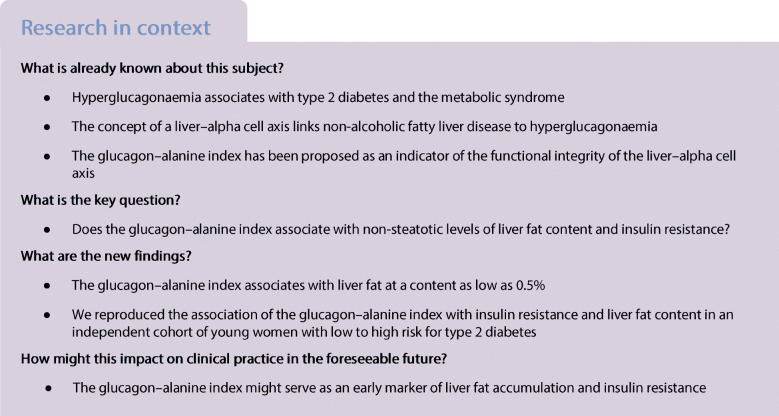



## Introduction

Increased plasma glucagon concentration (hyperglucagonaemia) has been suggested to play a crucial role in the development of type 2 diabetes. Indeed, inhibition of glucagon signalling by treatment with a glucagon receptor antagonist has shown favourable effects on glucose metabolism in individuals with type 2 diabetes [1, 2]. However, our previous finding that hyperglucagonaemia is not present in all individuals at high risk for type 2 diabetes suggests that hyperglucagonaemia might drive type 2 diabetes only in a subgroup of individuals [3]. Yet, the factors that drive hyperglucagonaemia in this subgroup have not been fully elucidated. Furthermore, many hyperglucagonaemic individuals exhibit characteristics of the metabolic syndrome [3], indicating that the metabolic syndrome might be closely related to hyperglucagonaemia.

The concept of a liver–alpha cell axis provides a potential causal link between the metabolic syndrome and hyperglucagonaemia [4, 5]. According to this concept, fat deposition in the liver, a hallmark of the metabolic syndrome [6], might impair hepatic glucagon signalling. Impaired hepatic glucagon signalling fosters hyperaminoacidaemia because glucagon regulates ureagenesis from amino acids [7]. Hyperaminoacidaemia, in turn, increases glucagon secretion from the alpha cell, which causes hyperglucagonaemia [4]. Hyperglucagonaemia may compensate for the increasing glucagon resistance and thereby restore amino acid metabolism. However, the action of glucagon on hepatic glucose production might be preserved, leading to increased hepatic glucose production [8]. Importantly, hyperglucagonaemia may aggravate the glucagon resistance of hepatocytes. Similar to insulin resistance, a vicious cycle develops in which the steadily rising hepatic glucose production eventually evolves into type 2 diabetes [5].

Evidence for this feedback cycle between the liver (amino acids) and alpha cells (glucagon) has previously been provided in participants of the ADDITION-PRO (‘Progression’ arm, nested within the Anglo–Danish–Dutch Study of Intensive Treatment In People with Screen Detected Diabetes in Primary Care study) study cohort [9]. In this context, the glucagon–alanine index was introduced as a surrogate marker for the functional status of the liver–alpha cell axis. The index is calculated as the product of fasting concentrations of plasma glucagon and plasma alanine and it was suggested that a higher index indicates functional impairments in the axis [9].

Hepatic steatosis might be causally involved in the impairment of the liver–alpha cell axis as individuals with non-alcoholic fatty liver disease (NAFLD) display elevated fasting glucagon and higher plasma levels of the sum of l-amino acids compared with individuals without the disease [10]. However, the possible contribution of lower levels of liver fat (i.e. lower than 5.5%) has not yet been studied.

In the present study, we examined the association of liver fat, at lower concentrations than those observed in NAFLD, and insulin resistance with the glucagon–alanine index. We hypothesised that liver fat content would associate with the glucagon–alanine index even at levels below the classification of NAFLD. This association would support the assumption that a functionally impaired liver–alpha cell axis is already present in the early pathogenesis of the metabolic syndrome and type 2 diabetes.

## Methods

### Cohort

**Fig. 1:**
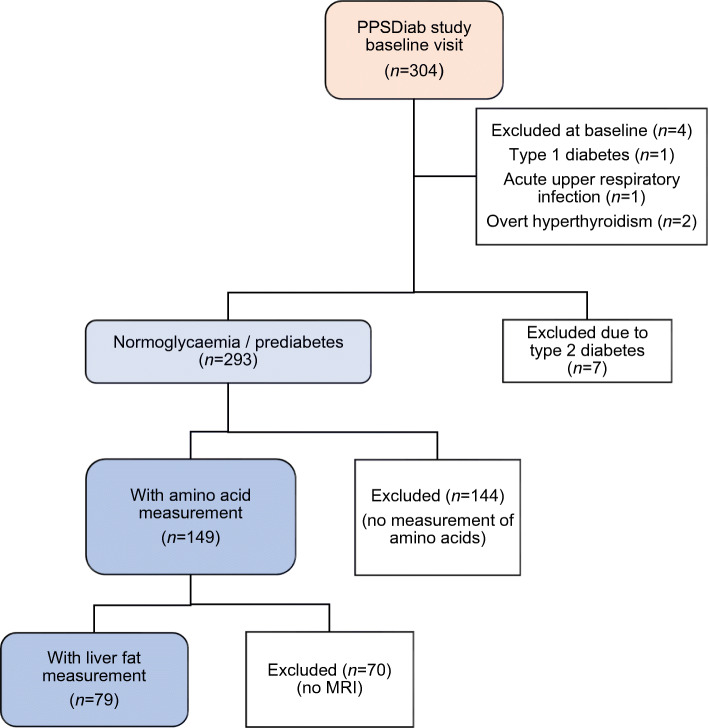
Flow chart for the PPSDiab study cohort

The present analyses were conducted on material obtained at the baseline visit of the Prediction, Prevention and Subclassification of Type 2 Diabetes (PPSDiab) study [11]. This study included 304 women 3–16 months after pregnancy. Participants were consecutively recruited from the diabetes centre and the obstetrics department of the University Hospital (Klinikum der Universität München) in Munich, Germany. Exclusion criteria for this study were alcohol or substance abuse, pre-pregnancy diabetes and chronic diseases requiring continuous medication (except for hypothyroidism [*n* = 52], bronchial asthma [*n* = 8], mild hypertension [*n* = 4], gastro-oesophageal reflux [*n* = 2] and history of pulmonary embolism resulting in rivaroxaban prophylaxis [*n* = 1]). Four women were excluded from the baseline visit of the PPSDiab study due to acute upper respiratory infection at the study visit (*n* = 1), overt hyperthyroidism (*n* = 2) or positive islet autoantibodies at baseline with diagnosis of type 1 diabetes during follow-up (*n* = 1) (Fig. 1).

For the present analyses, we further excluded women with type 2 diabetes diagnosis (*n* = 7) at the study visit to minimise possible bias from metabolic adaptions to overt diabetes. As previously described, measurement of plasma amino acids was only performed in the first half of the PPSDiab study cohort [12]. MRI for the determination of liver fat content was offered on a voluntary basis. Data on both plasma amino acids and liver fat content were available in 79 out of 293 (27%) participants without type 2 diabetes (Fig. 1). HOMA-IR and insulin sensitivity index (ISI) data were missing for one participant due to missing fasting insulin measurement.

All study participants provided written informed consent and the protocol was approved by the ethical review committee of the University Hospital in Munich (Ludwig-Maximilians-Universität, study ID 300-11). Detailed information on the study cohort is described in Rottenkolber et al [11].

### OGTT

We conducted an OGTT with a five-point measurement of plasma glucose and serum insulin as previously described [11]. For the diagnosis of diabetes, we used ADA criteria (fasting glucose ≥ 7.0 mmol/l and/or 2 h post-load glucose ≥11.1 mmol/l).

### Biochemical measurements and metabolomics

Glucagon and alanine levels were determined using frozen (−80°C) plasma samples collected in proteinase-stabilised tubes (BD P800; BD Biosciences, San Jose, CA, USA) after participants had fasted overnight. Plasma glucagon was measured by sandwich ELISA (catalogue no. 10-1271-01; Mercodia, Uppsala, Sweden). Alanine, as well as 20 other amino acids (arginine, asparagine, aspartate, citrulline, glutamine, glutamate, glycine, histidine, isoleucine, leucine, lysine, methionine, ornithine, phenylalanine, proline, serine, threonine, tryptophan, tyrosine and valine), were quantified by using targeted metabolomics (Absolute*IDQ*™ p180 Kit; Biocrates Life Sciences, Innsbruck, Austria) with LC-MS/MS. The measurement of plasma samples using this assay has been described in full detail previously [13]. Total amino acids represent the sum of all 21 l-amino acids, measured by the Absolute*IDQ*™ p180 Kit.

From the OGTT, plasma glucose was measured by a hexokinase method (Glucose HK Gen.3; Roche Diagnostics, Mannheim, Germany) and serum insulin by a chemiluminescent immunoassay (DiaSorin LIASON Systems, Saluggia, Italy). Fasting values of HDL-cholesterol and triacylglycerols were measured by enzymatic caloric test (Roche Diagnostics, Mannheim, Germany).

### Anthropometrics

Height and waist circumference were measured to the nearest 1 cm. Body mass was determined by a bioelectrical impedance analysis scale (Tanita BC-418; Tanita Corporation, Tokyo, Japan).

### MRI

Liver fat content was determined by MRI using an mDixon low-fat fraction map (3 Tesla System, Ingenia or Achieva; Philips Health Care, Hamburg, Germany). A detailed description of the MRI measurements is provided elsewhere [11].

### Calculations

To measure hepatic insulin resistance, HOMA-IR was calculated as fasting glucose (mmol/l) × fasting insulin (mmol/l) / 22.5. To determine peripheral insulin resistance, the ISI was calculated from the OGTT according to Matsuda and DeFronzo [14].

For the primary confirmatory analysis, the glucagon–alanine index was calculated as fasting glucagon × fasting alanine, as has previously been suggested [9]. Analogously, for secondary exploratory analyses, the glucagon–glutamate index and the glucagon–total amino acid index were calculated as fasting glucagon × fasting glutamate and fasting glucagon × fasting total amino acids, respectively.

### Statistical analysis

All metric and normally distributed variables are reported as mean ± SD; non-normally distributed variables are reported as median (first quartile–third quartile). Frequencies are presented as *n* (%). To address non-normality of the distribution of the glucagon–alanine index, liver fat content and HOMA-IR, all three variables were log-transformed for regression analyses. In addition, we compared liver fat quartiles using the Kruskal–Wallis test with Dwass, Steel, Critchlow-Fligner (DSCF) multiple comparison analysis [15]. The following analyses were conducted in all participants as well as stratified according to a liver fat content of ≤0.5% vs >0.5% (0.5% is used as proxy for the median of liver fat content in the cohort). Correlation analyses between glucagon, alanine, the glucagon–alanine index, liver fat and HOMA-IR were conducted using Spearman correlation coefficients (*ρ*). We also performed linear regression analyses with the glucagon–alanine index (log_e_-transformed) as the dependent variable and liver fat content or HOMA-IR (both log_e_-transformed) as independent variables adjusted for age and age plus HOMA-IR or liver fat, respectively. A *p* value <0.05 was considered statistically significant. All statistical calculations were performed using SAS statistical software package, version 9.4 (SAS Institute, Cary, NC, USA). Figures were created using Tableau 2020.3 (Tableau Software, Seattle, WA, USA).

## Results

Table 1 shows the baseline characteristics of the study cohort.

**Table 1:** Baseline characteristics of the study cohort

Characteristic	Measurement
N	79
Age, years	35.6 ± 4.0
Fasting glucagon, pmol/l	6.74 (4.76–8.28)
Fasting glucose, mmol/l	5.11 ± 0.41
2 h glucose, mmol/l	5.98 ± 1.26
Glucose status	
NGT	64 (81.01)
IFG	9 (11.39)
IGT	5 (6.33)
IFG+IGT	1 (1.27)
ISI (missing *n* = 1)	5.49 (3.68–8.43)
HOMA-IR (missing *n* = 1)	1.40 (0.93–2.30)
BMI, kg/m^2^	22.96 (21.26–26.85)
Waist circumference, cm (missing *n* = 2)	78 (72–86)
Systolic BP, mmHg	116 (110–123)
Diastolic BP, mmHg	71 (65–79)
Liver fat content, %	0.51 (0.19–1.27)
Triacylglycerols, μmol/l	723 (576–1028)
HDL-cholesterol, μmol/l	1604 (1267–1862)
Alanine, μmol/l	303.1 ± 75.0
Glucagon–alanine index	1.88 (1.29–2.59)

Values are presented as mean±SD or median (Q1–Q3). Frequencies are presented as *n* (%)

IFG, impaired fasting glucose; IGT, impaired glucose tolerance; NGT, normal glucose tolerance

**Fig. 2:**
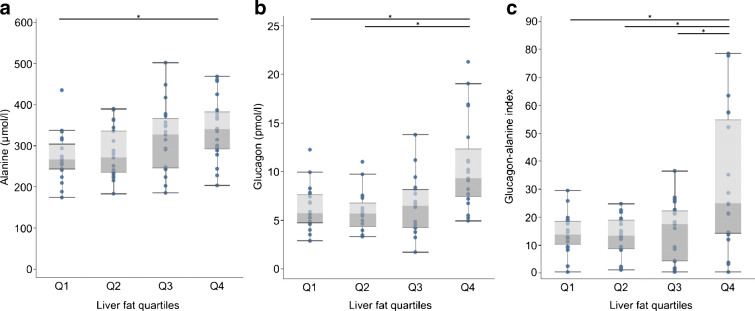
Fasting plasma alanine levels (**a**), fasting plasma glucagon levels (**b**) and glucagon–alanine index (**c**) stratified by liver fat quartiles. Boxes represent first quartile to median (dark grey) and median to third quartile (light grey). Whiskers depict 1.5-times the IQR. Group comparison by Kruskal–Wallis test with Dwass, Steel, Critchlow-Fligner post hoc test for multiple comparisons. *p < 0.05 for difference between groups

First, we compared fasting glucagon, alanine and the glucagon–alanine index between liver fat quartiles. Alanine continuously increased from quartile 1 to quartile 4 (Fig. 2a and ESM Table 1) whereas fasting glucagon and the glucagon–alanine index increased from quartile 2 to quartile 4 (Fig. 2b, c and electronic supplementary material [ESM] Table 1).

**Fig. 3:**
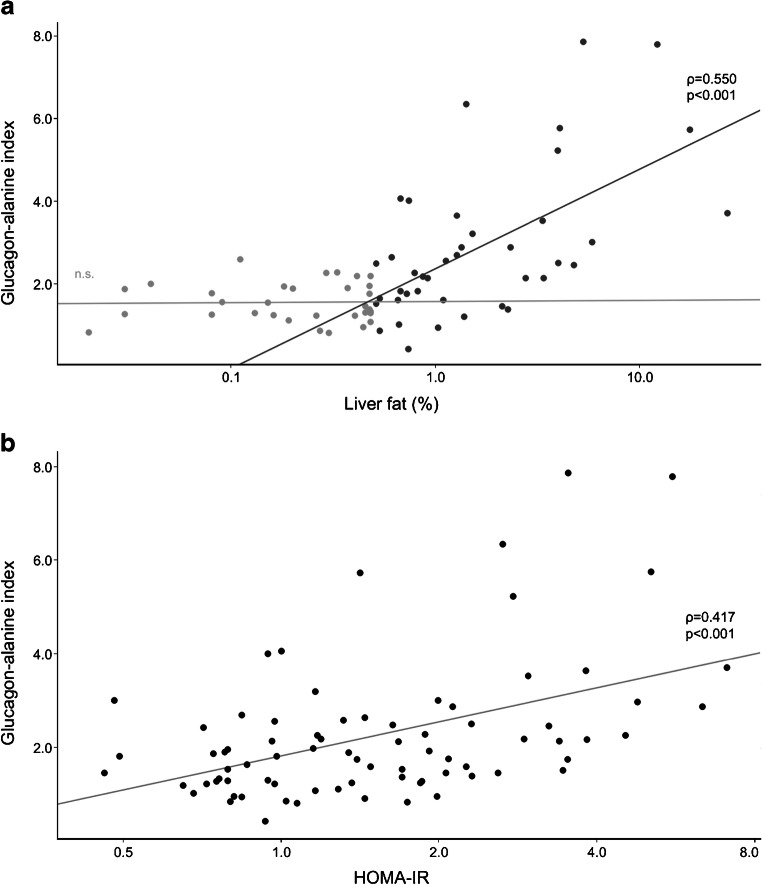
Association of the glucagon–alanine index with liver fat content (**a**) and HOMA-IR (**b**). Trend lines of crude models are shown together with Spearman correlation coefficients (*ρ*) and *p* values. The association between liver fat content and the glucagon–alanine index is shown as separate models for participants with liver fat content ≤0.5% (light grey dots; n.s.: non-significant association) and >0.5% (dark grey dots)

Alanine, glucagon, and the glucagon–alanine index correlated with liver fat content (Table 2). The correlation between the glucagon–alanine index and liver fat content began at around 0.5% fat, which was the median fat content in our cohort (Fig. 3a, Table 2 and ESM Fig. 1). Below 0.5%, the correlation was absent. In addition, the glucagon–alanine index correlated positively with HOMA-IR, fasting glucose and 2 h plasma glucose (Table 2 and ESM Fig. 1). The correlation with HOMA-IR was linear throughout the study cohort (Fig. 3b). Exploratory correlation analyses of other amino acids with liver fat content, glucagon and HOMA-IR are displayed in ESM Table 2. Of all the amino acids, glutamate showed the highest correlation to both glucagon and liver fat content, followed by the branched-chain amino acids.

**Table 2:** Spearman correlation of liver fat content and HOMA-IR with selected characteristics of the liver–alpha cell axis (fasting glucagon, alanine and glucagon–alanine index) for all participants (*n* = 79)

Variable	Fasting glucagon		Alanine		Glucagon–alanine index	
	*ρ*	*p* value	*ρ*	*p* value	*ρ*	*p* value
All participants (*n* = 79)						
Liver fat content	0.384	<0.001	0.372	<0.001	0.484	<0.001
HOMA-IR (missing *n* = 1)	0.373	<0.001	0.263	0.020	0.417	<0.001
Fasting glucose	0.187	0.099	0.205	0.070	0.240	0.033
2 h glucose	0.197	0.081	0.120	0.294	0.264	0.019
Participants with liver fat ≤0.5% (*n* = 39)						
Liver fat content	−0.151	0.357	0.026	0.877	−0.100	0.545
HOMA-IR	0.086	0.601	−0.129	0.433	−0.003	0.987
Participants with liver fat >0.5% (*n* = 40)						
Liver fat content	0.472	0.002	0.392	0.012	0.550	<0.001
HOMA-IR (missing *n* = 1)	0.391	0.014	0.334	0.038	0.429	0.006

In linear regression models, liver fat content still associated significantly with the glucagon–alanine index after an adjustment for age or age plus HOMA-IR, both in the whole study cohort as well as in the participants with a liver fat content of >0.5% (Table 3). Similarly, HOMA-IR associated with the glucagon–alanine index after adjustment for age or age plus liver fat content in all participants. However, in participants with a liver fat content of >0.5%, the association of HOMA-IR with the glucagon–alanine index was lost when liver fat content was added to the model (Table 3). No other glucagon–amino acid index associated as strongly as the glucagon–alanine index (ESM Tables 3, 4 show linear regression data for the glucagon–glutamate index and glucagon–total amino acid index, respectively).

**Table 3:** Linear regression analyses with glucagon–alanine index (log-transformed) as the dependent variable and liver fat content or HOMA-IR (both log-transformed) as independent variables

Participant group	Liver fat content			HOMA-IR (missing *n* = 1)		
	Adjusted *R*^2^	*β*	*p* value	Adjusted *R*^2^	*β*	*p* value
All participants (*n* = 79)						
Model 1^a^	0.112	0.367	0.001	0.188	0.457	<0.001
Model 2^b^	0.233	0.246	0.023			
Model 3^c^				0.233	0.378	<0.001
Liver fat ≤0.5% (*n* = 39)						
Model 1^a^	−0.012	−0.189	0.264	−0.042	0.074	0.659
Model 2^b^	−0.037	−0.184	0.286			
Model 3^c^				−0.037	0.057	0.736
Liver fat >0.5% (*n* = 40)						
Model 1^a^	0.280	0.563	<0.001	0.187	0.480	0.002
Model 2^b^	0.322	0.430	0.007			
Model 3^c^				0.322	0.283	0.069

Standardised regression coefficients, *β*, are shown

^a^Model 1: adjusted for age

^b^Model 2: adjusted for age and HOMA-IR

^c^Model 3: adjusted for age and liver fat

## Discussion

The present study provides evidence for a three-way association between liver fat content, the glucagon–alanine index and HOMA-IR. Thus, it supports the concept of a liver–alpha cell axis and suggests that alterations in this axis are already apparent at slightly increased amounts of liver fat content.

Our study substantiated the association between the liver–alpha cell axis and liver fat content. Studies in individuals with NAFLD and non-alcoholic steatohepatitis (NASH) illustrate that fat accumulation in the liver reduces the sensitivity of hepatocytes towards glucagon [16, 17]. One of glucagon’s actions on hepatocytes is the amplification of amino acid-induced urea synthesis [18]. Hence, a reduced glucagon sensitivity results in decreased urea formation from amino acids, leading to an increase in plasma amino acids [7, 19]. This rise in the levels of amino acids in turn stimulates glucagon secretion from the alpha cell, which promotes hyperglucagonaemia. Our analyses support this interrelation and further indicate that hepatic glucagon sensitivity might already be impaired at non-steatotic levels of liver fat, starting from 0.5%. Below this value, liver fat content and the glucagon–alanine index did not associate in our cohort. However, this may be related to the MRI measurement technique, which is the most sensitive among the non-invasive procedures but still not sufficiently sensitive for very low levels of fat [20].

Besides alanine, we also found glutamate to be involved in the liver–alpha cell axis (ESM Tables 2, 3). Glutamate activates AMPA/kainate receptors on the alpha cell, which increases glucagon secretion [21, 22]. Hence, a glucagon–glutamate index might also be suitable to represent the liver–alpha cell axis. In contrast to previous findings in individuals with and without NAFLD, total amino acids did not correlate with glucagon, liver fat or insulin resistance in our cohort of women with low levels of liver fat (ESM Table 2) and the association of a glucagon–total amino acid index was not superior to the glucagon–alanine index or the glucagon–glutamate index. Therefore, the determination of the glucagon–alanine index seems to be sufficient to characterise the status of the liver–alpha cell axis.

The extent to which the raised glucagon levels can compensate for the reduced glucagon sensitivity of hepatocytes regarding urea synthesis remains unknown. However, in the present study, the continuous association between liver fat content and the glucagon–alanine index points to an effective adaption of the liver–alpha cell axis to hepatic glucagon resistance in individuals without clinical steatosis (Fig. 3a).

Though adaption of the liver–alpha cell axis to hepatic glucagon resistance maintains amino acid turnover at a normal level, hyperglucagonaemia probably places a burden on glucose metabolism. In this context, our study revealed that insulin resistance and elevated plasma glucose levels associate with alterations in the liver–alpha cell axis (Table 2). Notably, the association of the glucagon–alanine index with insulin resistance was independent of liver fat content in the entire study cohort (Table 3). This independence was narrowly missing (*p* = 0.069) in the subgroup of participants with a liver fat content of >0.5% but we attribute this mainly to the smaller size of this subgroup. Previous research indicates that the action of glucagon on hepatic glucose production persists independent of a disturbance in hepatic glucagon sensitivity with regards to amino acid turnover [8]. In individuals with intact glucose metabolism, an increase in hepatic glucose production will be compensated for by enhanced insulin secretion. In the present study, the association of glucagon and the glucagon–alanine index with plasma glucose and insulin resistance (HOMA-IR) represented this compensation (Tables 2, 3). However, if insulin secretion and/or insulin sensitivity is not intact, hyperglucagonaemia is likely to aggravate the impaired glucose metabolism and finally lead to type 2 diabetes.

The present study supports the potential link between hepatic fat accumulation and insulin resistance via glucagon signalling despite the lack of a proven cause–effect relationship. Recent data in mice indicates that liver fat reduces the sensitivity of hepatocytes towards glucagon at both a transcriptional and a non-transcriptional level [23]. Conversely, pharmacological studies reveal that treatment with the glucagon receptor antagonist LY2409021 increased hepatic fat content in individuals with type 2 diabetes [2]. This might result from blockage of glucagon’s action on β-oxidation in the hepatocytes [24]. Hence, an increase in liver fat content might not only be the cause but also the result of glucagon resistance in hepatocytes. In any case, liver fat content seems to govern the sensitivity of hepatocytes towards glucagon. Thus, our results strongly support the concept of a liver–alpha cell axis.

Previous studies indicate that amino acids other than alanine may also be involved in the liver–alpha cell axis [7, 19]. The close associations of glutamate and the branched-chain amino acids with plasma glucagon that we observed support these previous results (ESM Table 2). However, no other glucagon–amino acid index performed better than the glucagon–alanine index in its associations with liver fat content and insulin resistance.

The strengths of our study include its cohort of young women at varying risk for type 2 diabetes who otherwise constitute an extremely homogeneous sample. By including only women with normoglycaemia, impaired fasting glucose or impaired glucose tolerance, we prevented possible bias resulting from sex differences and secondary changes in metabolism due to overt diabetes. Using MRI, we could quantify even low amounts of hepatic fat. The main weakness of our study is its cross-sectional design, which precludes the determination of a cause–effect relationship. Further, the homogeneity of the cohort regarding sex and age and the predominantly white ethnicity limits the transferability of our findings to the general public.

In conclusion, we reproduced the suggested association between liver fat, the glucagon–alanine index and insulin resistance in an independent study cohort of young women with low to high risk for type 2 diabetes. Our study supports the concept that even low levels of liver fat (up from 0.5%) are associated with the integrity of the liver–alpha cell axis, which probably affects glucose metabolism. To fully establish this concept, further mechanistic studies to examine the signalling at a molecular level are necessary. In addition, larger cohorts including men and more participants with an ethnicity other than white are required.

## Supplementary Information

ESM 1 (PDF 297 kb)

## Data Availability

The data that support the findings of this study are available from the corresponding author, AL, upon reasonable request.

## Abbreviations

ISI: Insulin sensitivity index
NAFLD: Non-alcoholic fatty liver disease
PPSDiab: Prediction, Prevention and Subclassification of Type 2 Diabetes
