# Supplementary material for: The liver–alpha cell axis associates with liver fat and insulin resistance: a validation study in women with non-steatotic liver fat levels
Source: Diabetologia. 2020 Dec 4;64(3):512–20. doi: 10.1007/s00125-020-05334-x (PMC7864806; doi:10.1007/s00125-020-05334-x)

Supplementary material

The liver–alpha cell axis associates with liver fat and insulin resistance: a validation study in women with non-steatotic liver fat levels

Christina Gar[123], Stefanie J Haschka[123], Stefanie Kern-Matschilles[123], Barbara Rauch[123], Vanessa Sacco[123], Cornelia Prehn[4], Jerzy Adamski[3456], Jochen Seissler[1], Nicolai J Wewer Albrechtsen[789], Jens J Holst[710]*, Andreas Lechner[123]*

[1] Diabetes Research Group, Department of Medicine IV, University Hospital, LMU Munich, Munich, Germany
[2] Clinical Cooperation Group Type 2 Diabetes, Helmholtz Zentrum München, Neuherberg, Germany
[3] German Center for Diabetes Research (DZD), Neuherberg, Germany
[4] Research Unit Molecular Endocrinology and Metabolism, Genome Analysis Center, Helmholtz Zentrum München, German Research Center for Environmental Health, Ingolstädter Landstraße 1, Neuherberg, Germany
[5] Department of Biochemistry, Yong Loo Lin School of Medicine, National University of Singapore, Singapore
[6] Chair of Experimental Genetics, Technical University of Munich, Freising-Weihenstephan, Germany
[7] Department of Biomedical Sciences, Faculty of Health and Medical Sciences, University of Copenhagen, Copenhagen, Denmark
[8] Department of Clinical Biochemistry, Rigshospitalet, Copenhagen, Denmark
[9] Novo Nordisk Foundation (NNF) Center for Protein Research, Faculty of Health and Medical Sciences, University of Copenhagen, Copenhagen, Denmark
[10] Novo Nordisk Foundation (NNF) Center for Basic Metabolic Research, Faculty of Health and Medical Sciences, University of Copenhagen, Copenhagen, Denmark

*Jens J. Holst and Andreas Lechner are joint senior authors

ESM Table 1: Median (Q1 - Q3) of fasting glucagon, alanine, and the glucagon-alanine index over the quartiles of liver fat.

	Q1	Q2	Q3	Q4
n	20	21	19	19
Glucagon-alanine index	1.54 (1.25-1.96)*	1.45 (1.22-1.95)*	1.83 (1.60-2.64)*	3.01 (2.14-5.74)
Glucagon, pmol/l	5.74 (4.72-7.62)*	5.67 (3.97-7.28)*	6.60 (4.25-8.13)	9.33 (7.22-13.54)
Alanine, µmol/l	267.5 (243.1-304.1)*	271.9 (235.2-335.9)	327.8 (243.1-373.9)	339.9 (288.1-387.3)
Glucagon-total amino acid index	14.72 (10.68-17.01)*	14.50 (9.14-16.33)*	15.63 (10.15-20.61)*	23.87 (15.40-44.90)
Total amino acids, µmol/l	2377.7 (2071.7-2608.3)	2458.2 (2214.2-2565.0)	2441.7 (2191.7-2524.9)	2664.7 (2240.9-2841.9)

Values are given as median (Q1-Q3). Group comparison with Kruskal-Wallis test and Dwass, Steel, Critchlow-Fligner post hoc test for multiple comparisons.
*Significant difference vs. Q4 ($p<0.05$).

ESM Table 2: Baseline distribution of other amino acids and spearman correlation coefficients (exploratory p-values) of the correlation with liver fat content, fasting glucagon, and HOMA-IR in all participants (n=79).

Amino acid	Mean plasma concentration (µmol/l)	Correlation with liver fat content		Correlation with glucagon		Correlation with HOMA_IR (missing n=1)	
	Mean±SD	ρ	p-value	ρ	p-value	ρ	p-value
Arginine	75.87±16.63	0.020	0.860	-0.049	0.671	-0.193	0.091
Asparagine	39.6±8.51	-0.027	0.811	0.056	0.624	-0.265	0.019*
Aspartate	5.92±1.89	0.116	0.308	0.277	0.013*	0.018	0.878
Citrulline	27.99±7.4	-0.098	0.391	-0.233	0.039*	-0.442	<0.001**
Glutamine	543.31±107.37	-0.043	0.707	-0.082	0.475	-0.199	0.080
Glutamate	43.75±20.63	0.311	0.005*	0.514	<0.001**	0.347	0.002**
Glycine	282.11±92.23	-0.127	0.265	-0.204	0.072	-0.345	0.002**
Histidine	74.82±12.13	0.110	0.333	0.159	0.161	-0.128	0.263
Isoleucine	58.48±10.88	0.253	0.025*	0.363	0.001**	0.364	0.001**
Leucine	117.72±20.16	0.292	0.009*	0.376	<0.001**	0.280	0.013*
Lysine	141.19±23.47	0.148	0.192	0.159	0.161	-0.015	0.893
Methionine	20.55±3.85	0.142	0.211	0.135	0.236	-0.117	0.308
Ornithine	40.55±9.66	0.061	0.591	-0.069	0.548	-0.209	0.066
Phenylalanine	52.42±8.18	0.285	0.011*	0.289	0.010*	0.128	0.266
Proline	137.31±30.52	0.243	0.031*	-0.010	0.931	0.209	0.067
Serine	103.41±24.38	-0.235	0.037*	-0.067	0.557	-0.260	0.021*
Threonine	100.04±26.13	0.037	0.745	-0.067	0.558	-0.050	0.666
Tryptophan	46.64±8.39	0.236	0.037*	0.185	0.103	0.084	0.466
Tyrosine	53.2±12.6	0.394	<0.001**	0.262	0.020*	0.290	0.010*
Valine	172.73±32.38	0.228	0.044*	0.414	<0.001**	0.282	0.013*
Total amino acids	2440.7±320.2	0.151	0.185	0.062	0.587	-0.060	0.604

*Correlations with exploratory significance (p<0.05). **Significant correlations after Bonferroni-correction for multiple testing.

ESM Table 3: Linear regression analyses. Dependent variable: glucagon-glutamate index (logarithmized); independent variables: liver fat or HOMA-IR (all logarithmized).

	Liver fat content			HOMA-IR (missing n=1)		
	Adjusted R^2	β	p-value	Adjusted R^2	β	p-value
All participants (n=79)						
Model 1[a]	0.056	0.239	0.033	0.188	0.433	<0.001
Model 2[b]	0.189	0.113	0.303			
Model 3[c]				0.189	0.397	<0.001
Participants with liver fat≤0.5% (n=39)						
Model 1[a]	0.141	-0.354	0.027	0.032	0.131	0.420
Model 2[b]	0.127	-0.345	0.033			
Model 3[c]				0.127	0.098	0.526
Participants with liver fat>0.5% (n=40)						
Model 1[a]	0.236	0.509	<0.001	0.187	0.467	0.003
Model 2[b]	0.279	0.366	0.024			
Model 3[c]				0.279	0.299	0.062

Standardised regression coefficients, β, are shown
[a]model 1: adjusted for age
[b]model 2: adjusted for age and HOMA-IR
[c]model 3: adjusted for age and liver fat

ESM Table 4: Linear regression analyses. Dependent variable: glucagon-total amino acid index (logarithmized); independent variables: liver fat or HOMA-IR (all logarithmized).

	Liver fat content			HOMA-IR (missing n=1)		
	Adjusted R^2	β	p-value	Adjusted R^2	β	p-value
All participants (n=79)						
Model 1[a]	0.066	0.299	0.008	0.105	0.358	0.001
Model 2[b]	0.133	0.209	0.067			
Model 3[c]				0.133	0.291	0.012
Liver fat ≤ 0.5% (n=39)						
Model 1[a]	0.002	-0.211	0.211	-0.042	-0.027	0.870
Model 2[b]	-0.024	-0.216	0.209			
Model 3[c]				-0.024	-0.048	0.774
Liver fat > 0.5% (n=40)						
Model 1[a]	0.273	0.557	<0.001	0.118	0.406	0.012
Model 2[b]	0.274	0.459	0.006			
Model 3[c]				0.274	0.195	0.218

Standardised regression coefficients, β, are shown
[a]model 1: adjusted for age
[b]model 2: adjusted for age and HOMA-IR
[c]model 3: adjusted for age and liver fat

ESM Fig. 1. Spearman correlation matrix of liver fat content, HOMA-IR, fasting glucagon, alanine, and glucagon-alanine index. Coloring represents the spearman correlation coefficient; size represents p-values of the correlations. HOMA-IR: homeostasis model assessment of insulin resistance.

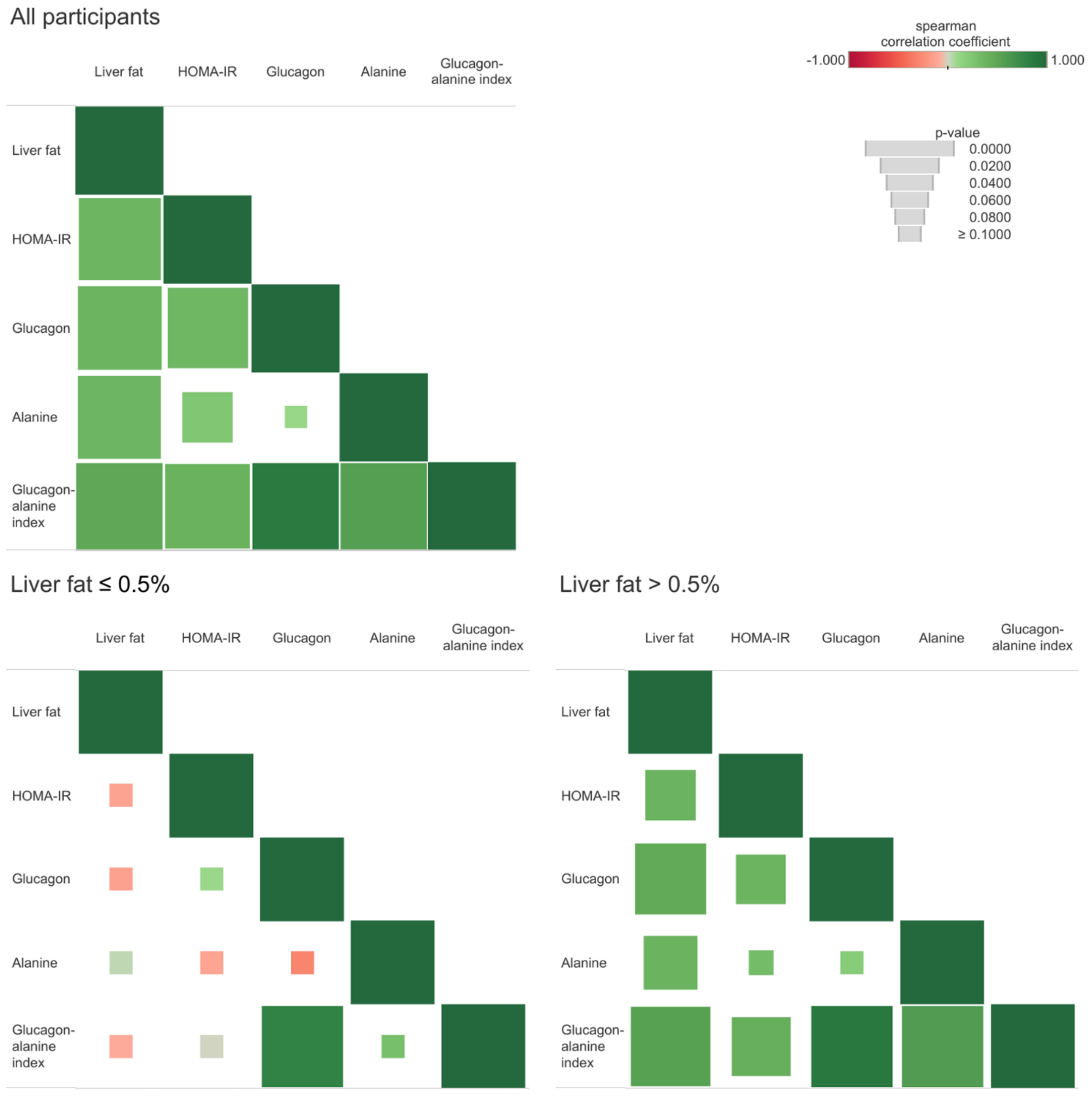

Supplement: Supplementary file 1 — (PDF 297 kb) [file 125_2020_5334_MOESM1_ESM.pdf]
